# Effect of supplemental feeding on metabolic profiles and meat quality in yaks: a non-targeted metabolomics approach

**DOI:** 10.1007/s44463-025-00027-8

**Published:** 2026-04-07

**Authors:** Meisong Wang, Yayu Huang, Jean-François Hocquette, Allan Degen, Zhi Liu, Rongzhen Zhong, Shujie Liu, Yang Xiang, Lizhuang Hao

**Affiliations:** 1 Key Laboratory of Plateau Grazing Animal Nutrition and Feed Science of Qinghai Province, Qinghai University, Xining, 810016 China; 2 PEGASE, INRAE, Institut Agro, 35590 Saint-Gilles, France; 3 INRAE, Clermont Auvergne, Université Clermont Auvergne, VetAgro Sup, UMR1213, Recherches sur les Herbivores, Theix, 63122 Saint Genès Champanelle, France; 4 Desert Animal Adaptations and Husbandry, Wyler Department of Dryland Agriculture, Blaustein Institutes for Desert Research, Ben-Gurion University of the Negev, 8410500 Beer Sheva, Israel; 5 College of Agriculture and Biotechnology, Hunan University of Humanities, Science and Technology, Loudi, 417000 China; 6 Changsha Xichu Information Technology Co., LTD, Changsha, 410000 China; 7 Jilin Province Feed Processing and Ruminant Precision Breeding Cross Regional Cooperation Technology Innovation Center, Jilin Provincial Laboratory of Grassland Farming, State Key Laboratory of Black Soils Conservation and Utilization, Northeast Institute of Geography and Agroecology, Chinese Academy of Sciences, Changchun, 130102 China

**Keywords:** Yak meat, Supplemental feeding, Non-targeted metabolomics, Meat quality, Biomarkers

## Abstract

The traditional grazing system of the Qinghai-Tibet Plateau faces challenges such as feed shortages, despite the abundance of pastures during the warm season. However, the impact of supplemental feeding on yak meat quality during this period still requires further investigation. A total of 30 male yaks (with similar genetic backgrounds, aged 2.5–3 years, weighing 94.56 ± 3.9 kg) were evenly and randomly assigned to two groups: the traditional grazing group (G) and the supplemental feeding group (SF). This study evaluated the effects of supplementation on yak meat quality and metabolic characteristics, aiming to identify effective dietary strategies to improve the physical and nutritional quality of yak meat. Non-targeted metabolomics (UHPLC-QE-MS) was used to analyze biomarkers of meat quality. Results revealed that the SF group exhibited superior meat quality, with a 39.6% reduction in shear strength, a 22.4% reduction in cooking loss, a 15% increase in PUFA/SFA ratios, and an 18% increase in essential amino acid content. Metabolomic profiling indicated distinct differences between the two groups, with the SF group demonstrating significant upregulation of beneficial metabolites (e.g., pyruvic acid, L-tyrosine, and eicosapentaenoic acid) and downregulation of harmful metabolites (e.g., sulfates). These changes improved protein turnover, lipid metabolism, and glycolytic activity, enhancing meat tenderness, flavor, and nutritional value. This study provides novel insights into the metabolic mechanisms underlying feed-induced quality changes, highlighting the practical value of supplemental feeding in overcoming the limitations of traditional grazing systems and reducing ecological pressure on grasslands.

**Supplementary Information:**

The online version contains supplementary material available at 10.1007/s44463-025-00027-8.

## Introduction

The yak (*Bos grunniens*), a unique domestic breed originating from the Qinghai-Tibet Plateau in China, is renowned for its association with the “roof of the world” (Wiener et al., 2003). With more than 14 million yaks, China accounts for approximately 92% of the global yak population, yielding an annual production of 300,000 tons of meat (Fan et al., 2019; Luo et al., 2006). Mainly found in the high-altitude and subalpine regions of the Qinghai-Tibet Plateau, generally at altitude above 3,000 m, yaks provide Tibetan herders with essential products such as milk, meat, wool, and hides (Luo et al., 2006).

Yaks are mainly raised by grazing in the Qinghai-Tibet Plateau region, where severe cold weather significantly affects vegetation, resulting in a very short growing season and prolonged dormancy periods. Poor grassland management and widespread rodent damage have exacerbated severe degradation of grasslands, leading to a rapid decline in forage quantity and quality (Li et al., 2022; Wei et al., 2022), resulting in reduced body weight of yaks from October to April (the cold season) (Long et al., 1999). Compared to other cattle breeds, yaks have lower productivity and inferior meat quality, which constrains the market share of yak meat products. The difficult winter slaughtering environment and the negative effects of cold weather on slaughtering operations and transportation hamper the immediate supply of fresh yak meat (Wen et al., 2015), making it impossible to meet market demand. From May to September (the warm season), with the increase in forage availability, the weight of yaks is expected to increase as this period which coincides with the grazing season. The pasture during the warm season appears sufficient to meet the nutritional needs of the yaks. However, studies have shown that yaks provided with concentrate feed exhibit better growth performance compared to the control group, particularly those around two years of age. Therefore, although the nutrition provided by the pasture during the warm season is adequate, the growth potential of yaks can still be further enhanced by supplementing concentrate feed during this period (Xue et al., 2021).

Supplementary feeding has been found to improve animal meat quality (Hossain et al., 2023; Volpelli et al., 2005; XU et al., 2020; Zhang et al., 2022b). Meat quality of free-grazed cattle is inferior to that of stall-fed yaks in terms of color, shear force, beneficial fatty acid ratio, and composition (Ma et al., 2021; XU et al., 2020). It has been reported that the nutritional value of warm-season forages can only satisfy the essential growth needs of yaks, concentrate supplementation during this period can further enhance their growth and improve meat quality (Xue et al., 2005). This study investigates the effects of supplementary feeding during warm-season grazing on yak meat quality. The mechanisms behind these effects remain unclear. Metabolomics, the comprehensive study of small molecules within biological organisms, has become a technique for exploring changes in metabolic response under environmental influences, focusing on molecules generally smaller than 1500 kDa (Kuehnbaum & Britz-Mckibbin, 2013; Lao et al., 2009). It has become a powerful tool in animal feeding studies, offering insights into the ultimate outcomes of nutritional interventions (Nicholson et al., 1999; Noguchi et al., 2003; Rezzi et al., 2007; Tang et al., 2006). Meat metabolites, as key phenotypic components, are significantly associated with meat quality traits (Muroya, 2023). Therefore, analyzing changes in these key metabolites could help reveal the underlying mechanisms through which different processes lead to variations in meat quality. The effects of various intrinsic and extrinsic factors on meat metabolomics have been assessed, including muscle type (Setyabrata et al., 2023), breed (Gómez et al., 2020), packaging methods (Samuelsson et al., 2022), aging conditions (Kim et al., 2020; Setyabrata et al., 2021), and feeding conditions (Stella et al., 2006). King et al., (King et al., 2019) analyzed molecular differences between tender and tough beef steaks, suggesting that the concentrations of glucose, glucose-6-phosphate, and malic acid could serve as biomarkers to distinguish differences in beef tenderness. Furthermore, untargeted metabolomics combined with ultra-high-performance liquid chromatography-tandem mass spectrometry (UHPLC-QTOF-MS) detected differential metabolites in Tibetan sheep meat subjected to different feeding patterns. By associating these metabolites with meat quality traits, four potential biomarkers with predictive value were identified, offering a new scientific basis for meat quality assessment (Zhang et al., 2022b). In summary, metabolomics is expected to offer new insights into the mechanisms underlying meat quality changes in yaks under supplemental feeding conditions.

The present study hypothesizes that a cereal-rich supplement could affect meat quality and metabolism of yaks, mainly with regard to muscle metabolism. Using untargeted metabolomics technology UHPLC-QE-MS, differential metabolites in the *longissimus lumborum* (LL) muscle of yaks could be identified. In addition, Kyoto Encyclopedia of Genes and Genomes (KEGG) pathway analysis was used to reveal the impact of feeding conditions on metabolic changes.

## Materials and methods

### Animals, slaughter, and meat samples

The detection methods and related data for the characterization of yak meat quality in this study originate from the previously published paper of our research team, and the relevant citation has been authorized by the original journal (Wang et al., 2025). All experiments were conducted in accordance with the National Laboratory Animal Welfare Guidelines (2006–398) and were approved by the Animal Use Committee of the Academy of Science and Veterinary Medicine of Qinghai University (Approval No. QHU20150301).

Since the primary focus of this study was to evaluate the effect of a cereal-rich supplement on the diet of yaks, considering also constraints in resources and experimental conditions, the experiment involved 30 male yaks with similar characteristics (Similar genetic background, aged 2.5–3 years, weight 94.56 ± 3.9 kg). These yaks were randomly assigned to either a supplemental feeding group (SF, n = 15) or a traditional grazing group (G, n = 15). In this study, the Traditional Grazing group (G) refers to yaks that are raised through natural grazing without any supplemental feeding.​ This method relies solely on the nutrients available in the natural pasture, without providing additional feed supplements. The study was conducted during the warm season (June to October) in the high-altitude meadows of Saierlong Township, Henan Mongolian Autonomous County, Qinghai Province, situated in the heart of the Qinghai-Tibet Plateau (N 34°34′44.01″ to 34°35′21.68″, E 101°49′44.12″ to 101°50′47.06″). The yaks in both the supplemental feeding and grazing groups were grazed separately in two adjacent meadows with identical grass compositions. Both groups were allowed to graze daily from 7:30 am to 7:30 pm. The yaks in the supplemental feeding group were given 750 g of pelleted feed before and after grazing, respectively (see Table 1). All yaks had free access to water. After a 10-day adaptation period, all yaks participated in a formal 120-day experiment. The management and slaughtering of the yaks were carried out in accordance with the DB63/T 1740—2019 'Yak Full-Cycle Breeding Management Technical Specification’ and DB63/T 1785–2020 'Yak Slaughtering Technical Regulations.' These standards respectively regulate the entire management process of yaks from breeding to fattening, as well as the hygiene and animal welfare requirements during slaughter operations, ensuring the standardization of the research process and the reliability of the results. At the end of the feeding period, following animal welfare principles, 30 yaks underwent a 12-h fasting period and a 2-h water deprivation before being transported to the slaughterhouse. Samples were then taken from the *longissimus lumborum* muscle near the 12-13^th^ ribs of each carcass. Professionals conducted the slaughter and sampling according to standardized procedures. All collected samples were immediately immersed in liquid nitrogen, transported to the laboratory, and stored in a −80 °C freezer for subsequent analysis. However, considering the experimental costs and the expected accuracy of the results, we conducted an auxiliary omics analysis on 12 yaks, with six animals in each group.

**Table 1:** Formulation of complementary concentrated feeds and their nutrient content (DM basis)

Items	Content (%)
Ingredient composition	
Corn	44.90
Wheat bran	25.25
Double low rapeseed meal	12.65
Heat-treated rapeseed	12.00
Stone powder	2.50
Premix1	1.00
Bentonite	1.00
Salt	0.60
Calcium hydrogen phosphate	0.10
Nutritional composition(dry matter basis)	
Dry matter (%)	93.61
Crude protein (%)	18.15
Ether extract (%)	8.76
Neutral detergent fiber (%)	20.5
Acid detergent fiber (%)	8.25
Ca (%)	1.21
P (%)	0.79
NaCl (%)	0.66
Gross energy (MJ/kg)	18.38
Dry matter digestibility (%)	86.71
Metabolizable energy (MJ/kg)	13.07

### Meat quality analysis

#### Physical quality analysis

Analysis of the physical quality of meat, including pH, color, Warner–Bratzler shear force (WBSF), cooking loss, and drip loss, were conducted for all samples. Except for the pH value measured 45 min after slaughtering, all other indicators were measured using conventional methods 24 h postmortem.

The pH was measured by inserting a portable pH meter (FE20, Mettler Toledo, Copenhagen, Denmark) 2 cm into the meat sample. A colorimeter (CR-400, Konica Minolta Sensing Americas, Ramsey, USA) was pressed against the muscle surface without gaps, and readings for brightness (L*), redness (a*), and yellowness (b*) were recorded after stabilization. Additionally, the meat sample was wrapped and sealed in a polyethylene bag, cooked in a water bath at 80 °C for 40 min, then cooled to room temperature. Surface moisture was blotted with filter paper, and the meat was cut along the muscle fibers into cylinders (1 cm × 1 cm × 3 cm) for measuring tenderness (tenderness meter, RH-N50, Baoding Tianhua Measurement Instrument Co., Ltd., Baoding, China). Cooking loss was determined by cooking meat samples (1 cm × 1 cm × 2 cm) in an 85 °C water bath for 20 min, with the loss calculated as the percentage of weight change relative to the initial sample weight. Similarly, drip loss was calculated as the percentage of water loss relative to the initial weight of the meat sample after hanging at 4 °C for 24 h.

#### Nutritional quality analysis

The recognized AOAC methods (AOAC, 2006) were used to determine the moisture (AOAC 950.46), crude protein (AOAC 928.08), crude fat (AOAC 991.36), total ash (AOAC 920.153), and cholesterol contents (AOAC 944.10) in the *Longissimus lumborum* (LL).

Moisture content was determined after complete drying of meat samples in an oven at a temperature of 103 ± 2 °C. The crude protein content was determined by the Kjeldahl method and the crude fat content via the Soxhlet extraction method. Total Ash content was determined after incineration of the samples in a crucible at 550 ± 25℃ for 4 h. Cholesterol content was determined using gas chromatography after direct saponification.

Amino acids and fatty acids in meat samples were analyzed using UHPLC and GC–MS (Gas Chromatography–Mass Spectrometry), respectively. UHPLC, used for liquid-phase analysis, used the Agilent 1290 Infinity system with a mobile phase system consisting of A (25 mM ammonium formate and 0.08% formic acid in water) and B (0.1% formic acid acetonitrile) solutions. GC–MS, a gas-phase analysis tool, used the Agilent 7890/5975C system with separation on a capillary column (30 m × 0.25 mm ID × 0.25 μm, DB-WAX). The experimental procedures were conducted following the method outlined by Zhang et al. (2022b).

### Untargeted metabolomics profiling

#### Extraction of metabolites

An aliquot of 100 μL of sample was mixed with 300 μL methanol and 20 μL internal standard (2-Chloro-L-phenylalanine, CAS: 103,616–89-3, purity ≥ 98%). The mixture was vortexed for 30 s using a vortex mixer (VORTEX-5, Qilinbei Instrument Manufacturing Co., Ltd.). The sample was then subjected to ultrasonic extraction for 5 min in an ice-water bath using an ultrasonic device (PS-60AL, Shenzhen Redebang Electronics Co., Ltd., China), followed by 2 h of static incubation at −20℃. Afterward, the sample was centrifuged at 13,000 rpm for 15 min at 4℃ using a centrifuge (Thermo Scientific Heraeus Fresco17). A 200 μL aliquot of the supernatant was transferred to a 2 mL injection vial for LC–MS analysis, which was conducted using an ultra-high-performance liquid chromatography system (Agilent 1290 UHPLC, Agilent) and a high-resolution mass spectrometer (Q Exactive Orbitrap, Thermo Fisher Scientific, USA).

#### LC–MS/MS analysis

LC–MS/MS analyses were performed using an UHPLC system (1290, Agilent Technologies) with a UPLC HSS T3 column (2.1 mm × 100 mm, 1.8 μm) coupled to Q Exactive (Orbitrap MS, Thermo). The mobile phase A was 0.1% formic acid in water for positive ions, and 5 mmol/L ammonium acetate in water for negative ions, and the mobile phase B was acetonitrile. The elution gradient was set as follows: 0 min, 1% B; 1 min, 1% B; 8 min, 99% B; 10 min, 99% B; 10.1 min, 1% B; 12 min, 1% B. The flow rate was 0.5 mL/min. The injection volume was 2 μL. The QE mass spectrometer was used for its ability to acquire MS/MS spectra on an information-dependent basis (IDA) during an LC/MS experiment. In this mode, the acquisition software (Xcalibur 4.0.27, Thermo) continuously evaluates the full scan survey MS data as it collects and triggers the acquisition of MS/MS spectra depending on preselected criteria. ESI source conditions were set as following: Sheath gas flow rate as 45 Arb, Aux gas flow rate as 15Arb, Capillary temperature 320 °C, Full ms resolution as 70,000, MS/MS resolution as 17,500, Collision energy as 20/40/60 eV in NCE model, Spray Voltage as 3.8 kV (positive) or −3.1 kV (negative), respectively.

### Statistics analysis

Quantitative data were subjected to one-way analysis of variance (ANOVA) using SPSS software (version 22.0) with one factor (the feeding system). For non-targeted metabolomics data, MS raw data (.raw) files were converted to the mzML format using ProteoWizard, and then processed with the R package XCMS (version 3.2). The preprocessing results generated a data matrix containing retention time (RT), mass-to-charge ratio (m/z) values, and peak intensity. After XCMS data processing, peak annotation was performed using OSI-SMMS software (version 1.0, Dalian Chem Data Solution Information Technology Co. Ltd.) with an in-house MS/MS database. In addition, multivariate statistical analysis was performed using SIMCA software (version 14.0), including principal component analysis (PCA), orthogonal partial least squares discriminant analysis (OPLS-DA), 200 permutation tests for OPLS-DA, and analysis of variable importance in projection (VIP) plots. T-tests were performed based on VIP values ≥ 1 and P ≤ 0.05 to filter out significantly different metabolites. The LDA algorithm were implemented in MATLAB, (2016). Metabolic pathway analysis was conducted through KEGG (Kyoto Encyclopedia of Genes and Genomes). Use the pheatmap package and the EnhancedVolcano package in R (version 4.0) to generate a clustering heatmap and a volcano plot, respectively.

A one-way analysis of variance (ANOVA) was conducted to evaluate differences between groups and assess the impact of these differences on meat classification (Bewick et al., 2004). However, a single variable often does not allow for a full discrimination between similar groups. Therefore, several multivariate analysis methods, such as principal component analysis (PCA) and orthogonal partial least squares discriminant analysis (OPLS-DA), were used. Unsupervised principal component analysis (PCA) was then applied for dimensionality reduction and denoising of the raw data, calculating the Mahalanobis distance between each pair of samples and clustering based on the nearest neighbor principle (Köhn & Hubert, 2014). The unsupervised model was transformed into a supervised model (OPLS-DA) to achieve more satisfactory discrimination of the sample groups. Based on prior knowledge of sample categories, OPLS-DA discriminates the target objects by decomposing and fitting observed or measured feature variables. This method aims to find a linear regression model that optimally classifies samples by projecting observed and predicted variables into a new space (Ballabio et al., 2014). To validate the accuracy of the OPLS-DA model developed through 200 random cycles, leave-one-out cross-validation was performed. The key advantage of leave-one-out cross-validation is that it uses as many samples as possible in each iteration for training, and the results of each calculation are consistent (Browne, 2000). Linear Discriminant Analysis (LDA) is a supervised dimensionality reduction method, which aims to find a projection direction that maximizes the between-class variance and minimizes the within-class variance after projection. This mapping can be used both for data preprocessing and directly as a classifier.

## Results and discussion

### Feed intake and conversion efficiency

The results of feed intake and feed efficiency for both groups are shown in Table 2. Supplementation significantly increased the total feed intake of the yaks while also significantly reducing their reliance on natural pasture (*P* < 0.01). Specifically, compared to the grazing group (G), yaks in the supplement group (SF) consumed 0.38 kg less grass per day. This finding suggests that supplementation can effectively alleviate grazing pressure on natural grasslands, which is crucial for sustainable grassland management on the Qinghai-Tibet Plateau. In terms of daily weight gain, animals of the SF group were significantly heavier than those of the G group (*P* < 0.01), with the SF group achieving a daily weight gain 2.01-fold higher of that the G group. The feed-to-gain ratio, calculated from daily weight gain and feed intake, was significantly lower in the SF group than in the G group (*P* < 0.01).

**Table 2:** Effect of yak supplementary feeding on its feed intake and feed efficiency grazing on alpine grassland in warm season

Parameters	Groups		SEM	P-value
	Grazing (G)	Supplementary feeding (SF)		
Forage intake (kg/d)	3.86*	3.48	0.067	0.01
Additional feed amount (kg/d)		1.5		NS
Total intake (kg/d)	3.86	4.98**	O.12	< 0.001
Daily weight gain (g/d)	332	669.33**	33.817	< 0.001
Feed conversion efficiency (Feed-to-gain ratio)	12.08	7.89**	0.487	< 0.001

^*^ Significant at *P* < 0.05

^**^ Significant at *P* < 0.01

*Ns* Not significant at *P* > 0.05

*SEM* Standard Error of the Mean

### Yak meat quality according to two feeding patterns

#### Physical quality of meat

As shown in the yak meat quality parameters in Table 3, the SF group showed a significantly lower meat pH at both 45 min (*P* < 0.05) and 24 h (*P* < 0.001) post-slaughter compared to the G group. This lower pH is beneficial for meat quality as it prevents the formation of dark, firm, and dry (DFD) meat, which is often less preferred by consumers. It is worth noting that both the G group and the SF group meet the RFN (Red, Firm, Non-exudative) beef standards, with an initial pH value of 6.0–6.8 and a final pH value of 5.5–6.1. This difference in pH values might be attributed to higher glycogen reserves in the muscles of yaks under additional feeding, leading to increased lactic acid production *postmortem* (Antonelo et al., 2022; Barrasso et al., 2022). In the present study, the lower meat pH value in the SF group compared to the G group may be associated with variations in the rate of glycogen breakdown in the meat (Zi-Xuan et al., 2018). However, the pH values of the meat in both the G group and the SF group remained within the normal range. Consequently, yak meat produced through supplementary feeding is more likely to meet consumers’ expectations (Jelic Milkovic et al., 2023).

**Table 3:** Effects of different feeding methods on the quality of yak meat(Wang et al., 2025)

Parameters	Groups		SEM	P-value
	Grazing (G)	Supplementary feeding (SF)		
pH (45 min post mortem)	6.26 ± 0.22	6.08 ± 0.15*	0.038	0.011
pH (24 h post mortem)	5.39 ± 0.16	5.18 ± 0.13**	0.033	< 0.001
L*	35.06 ± 1.3	35.89 ± 1.87 NS	0.299	0.170
a*	18.03 ± 1.48	18.51 ± 1.17 NS	0.244	0.336
b*	9.57 ± 1.39	9.31 ± 1.22 NS	0.236	0.593
Cooking loss (%)	29.22 ± 2.69	26.85 ± 1.5b**	0.448	0.006
drip loss (%)	21.3 ± 1.81	19.26 ± 1.25**	0.338	0.001
Shear force (Kg)	6.77 ± 0.67	4.09 ± 0.56b**	0.273	< 0.001
Water (%)	75.53 ± 2.57	75.07 ± 2.02 NS	0.417	0.594
Dry matter (%)	24.27 ± 2.57	24.93 ± 2.02 NS	0.417	0.594
Total amino acid content (TAA, g/100 g)	13.25 ± 0.46	14.14 ± 0.65**	0.131	< 0.001
Essential amino acid content (EAA, g/100 g)	5.19 ± 0.18	6.03 ± 0.35**	0.092	< 0.001
SFA (%)	43.25 ± 1.54	39.22 ± 1.62**	0.469	< 0.001
MUFA (%)	46.66 ± 2.17	47.11 ± 2.8 NS	0.452	0.627
PUFA (%)	11.49 ± 0.45	13.74 ± 0.34**	0.220	< 0.001
PUFA/SFA	0.27	0.35**	0.008	< 0.001
n-3 (%)	2.81 ± 0.13	5.31 ± 2.19**	0.234	< 0.001
n-6 (%)	8.08 ± 0.33	7.64 ± 3.21**	0.074	< 0.001
n-6/n-3 (%)	2.88	1.44**	0.135	< 0.001
Atherogenic Index (AI) (%)	0.49 ± 0.03**	0.36 ± 0.03	0.013	< 0.001
Crude Protein (%)	22.18 ± 1.67	22.62 ± 2.11 NS	0.344	0.530
Crude Fat (%)	3.56 ± 0.84	6.17 ± 0.53**	0.273	< 0.001
Ash Content (%)	3.56 ± 0.84	1.57 ± 0.29 NS	0.058	0.064
Cholesterol (mg/100 g)	95.78 ± 7.34	83.91 ± 8.06**	1.768	< 0.001

^*^ Significant at *P* < 0.05

^**^ Significant at *P* < 0.01

NS Not significant at *P* > 0.05

SFA (Saturated Fatty Acids) = (C22:0 + C10:0 + C16:0 + C15:0 + C17:0 + C14:0 + C13:0 + C11:0 + C8:0 + C21:0 + C18:0 + C12:0 + C24:0 + C23:0 + C20:0)

MUFA (Monounsaturated Fatty Acids) = (C18:1t n-9 + C17:1n-7 + C16:1n-7 + C15:1n-5 + C22:1n-9 + C18:1n-9 + C20:1n-9 + C14:1n-5 + C24:1n-9)

PUFA (Polyunsaturated Fatty Acids) = (C20:2n-6 + C22:4n-6 + C20:3n-3 + C18:2n-6 + C22:2n-6 + C22:6n-3 + C18:3n-6 + C20:3n-6 + C18:3n-3 + C18:2 t t n-6 + C20:4n-6 + C22:5n-3 + C20:5n-3 + C22:5n-6)

n-3 = (C20:3n-3 + C22:6n-3 + C18:3n-3 + C22:5n-3 + C20:5n-3)

n-6 = (C20:2n-6 + C22:4n-6 + C18:2n-6 + C22:2n-6 + C18:3n-6 + C20:3n-6 + C18:2 t t n-6 + C20:4n-6 + C22:5n-6)

AI (atherogenicity index) = [(C12:0 + 4*C14:0 + C16:0)/(MUFA + PUFA)](Ulbricht & Southgate, 1991)

In terms of other quality aspects, the SF group shows a significant advantage over the G group. Indeed, meat from the SF group exhibits a significant reduction in cooking loss (*P* < 0.01), drip loss (*P* < 0.01), and especially in shear force, decreasing from 6.77 kg to 4.09 kg (*P* < 0.001). This substantial difference highlights a significant improvement in meat tenderness by supplementary feeding method. Tenderness, as one of the core characteristics of beef quality, plays a crucial role not only in the sensory experience of meat but also influences processing characteristics, making the product more favored by consumers (Miller, 2020). Our study, by supplementing specific high-quality feed, has reduced the shear force of yak meat, thereby improving its tenderness, which aligns with findings from previous studies (Ma et al., 2024). This may be attributed to supplementary feeding providing yaks with more abundant and balanced nutrition, promoting meat growth and development, enhancing the integrity and toughness of muscle cell membranes, and expanding intracellular microstructures, the combined effect of these factors contributes to the improvement of muscle structure, reduction of connective tissue components, and enhancement of cell membrane resistance to degradation, thereby reducing fiber breakdown during cooking, improving meat tenderness, and ultimately leading to a decrease in shear force (Yao et al., 2022). Some research also indicate that supplementary feeding can introduce protective fats while increasing fat deposition between muscles, thereby improving the texture, juiciness, flavor, and tenderness of meat (Frank et al., 2016b; Hwang & Joo, 2017; Jung et al., 2016).

#### Nutritional quality of meat

Regarding fatty acid composition, supplementation did not significantly affect monounsaturated fatty acids (MUFA) proportion (P > 0.05). However, it significantly decreased saturated fatty acids proportion (SFA), n-6/n-3 ratio, and Atherogenic Index (AI), while increasing polyunsaturated fatty acids (PUFA), PUFA/SFA ratio, and n-3 polyunsaturated fatty acids (*P* < 0.001). These changes are noteworthy as higher levels of n-3 PUFA and a lower n-6/n-3 ratio have been linked to improved cardiovascular health and reduced risk of chronic diseases (Roszkos et al., 2021).Thus, supplementation enhances the nutritional value of yak meat for health-conscious consumers. In our study, adding heated rapeseed to feed may explain the elevated levels of polyunsaturated fatty acids (PUFA) in the muscle of the SF group.

Additionally, supplementary feeding markedly increased total amino acid content (TAA) in beef (*P* < 0.001), with a particular emphasis on essential amino acids (EAA), crucial for the human body. Indeed, the human body cannot synthesize them independently or produces them in insufficient amounts, necessitating their acquisition through dietary intake. The Joint Expert Committee of FAO/WHO/UNU specifies that, for dietary protein to be deemed ideal, the ratios of EAA/TAA and EAA/NEAA (Essential to Non-Essential amino acids ratio) should both surpass 40% and 60%, respectively (Organization, 2007). The results indicated that supplementing with a specific diet can elevate the ratios of EAA/TAA and EAA/NEAA from 39.2% and 64.9% to 42.6% and 74.6%, respectively, aligning more closely with the amino acid patterns essential for human dietary intake.

Following supplementation, there were no changes in the moisture, crude protein, and crude ash content of yak meat (*P* > 0.05), yet a slight downward trend was observed in crude ash content, decreasing from 3.56% to 1.57% (*P* > 0.05). This suggests that supplementation did not notably affect the majority of crude nutritional components, mainly contributing to increased productivity. Nevertheless, in this study, the fat content was substantially increased (*P* < 0.001) from 3.56% to 6.17%. This can be attributed to the incorporation of feeds with higher fat content in the supplementation (Raes et al., 2004; Xiong et al., 2022; Zhang et al., 2025). Notably, the fat content not only supplies essential fatty acids and fat-soluble vitamins but also plays a role in the development of the so-called “marbling” in the meat (Yu et al., 2023). Research by Frank et al. (2016a) showed that in grilled beef higher marbling levels (for instance for Wagyu beef) enhance flavor, dairy fat aroma, and sweetness, while reducing sourness and astringency. Additionally, higher marbling may increase tenderness and juiciness.

Since the confirmation of the correlation between cholesterol concentration in rabbit plasma and atherosclerosis and cardiovascular diseases in 1913 (Vance & Bosch, 2000), cholesterol levels in food have received increasing attention. As a result, cholesterol has also become a crucial element in examining meat and poultry products. The findings of this study indicated that supplementing grazing yaks with feed containing matured double-low rapeseed significantly reduced cholesterol content and atherosclerosis index (AI) in meat (*P* < 0.001). This aligns with findings from various studies (Dus-Zuchowska et al., 2019; Najera et al., 2017). This could be attributed to the presence of unsaturated fatty acids in the supplemented diet, which facilitates the digestion and absorption of cholesterol in the digestive tract of ruminants, while also reducing its accumulation in the muscles (Libera et al., 2020; Pourshahidi et al., 2020; Reece et al., 2015; Roszkos et al., 2021).

### Metabolite levels in yak meat according to two feeding patterns

**Fig. 1:**
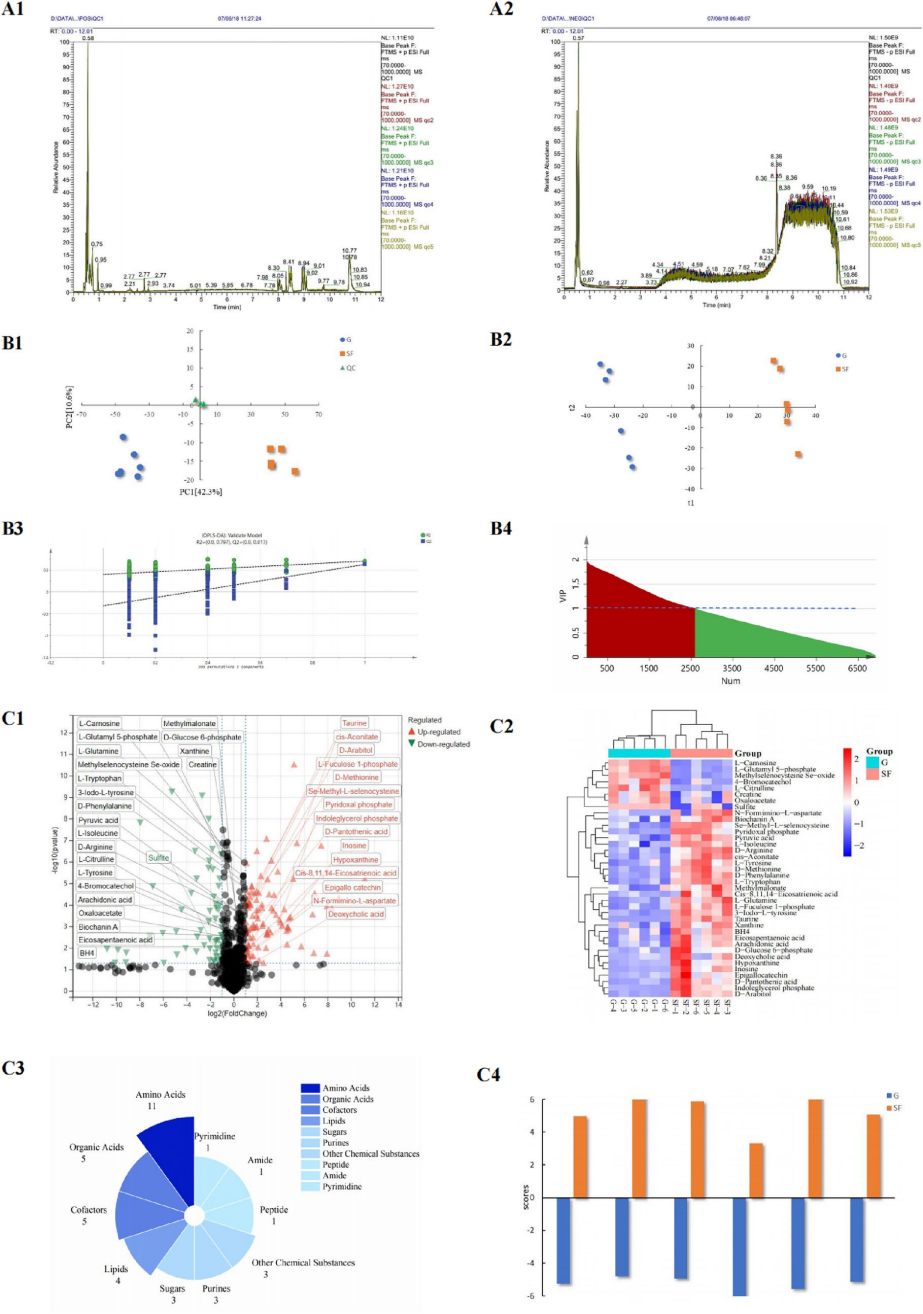
Metabolites of yak meat in two feeding patterns: positive and negative ion base peak chromatograms (BPC) of samples (Figures **B1** and **A3**); PCA score plots of all detected metabolites in positive and negative ion patterns (**B1**), OPLS-DA score plots (**B2**), results of permutation test and VIP plots for OPLS-DA (**B3**, **B4**); volcano plot analysis of 38 annotated differential metabolites (**C1**) (OPLS-DA VIP > 1 and P-value < 0.05), heatmap of 38 significantly different metabolites (**C2**), distribution analysis of 38 significantly different metabolite types (**C3**), and LDA discrimination results for yak meat samples fed according to two different patterns (**C4**)

In the positive and negative ion groups, 4008 and 2925 metabolites were detected respectively (Tables S1 and S2). To assess distinctions in the metabolomic profiles of yaks under two feeding patterns, as depicted in Fig. 1 A1 and A2, the retention time and signal intensity of the chromatographic peaks in the BPC (Base Peak Chromatogram) of QC samples exhibited substantial overlap, indicating favorable instrument stability. An unsupervised principal component analysis (PCA) method was applied to obtain an overview of the phenotypic differences in metabolites. The PCA score plot showed tight clustering and distribution of QC samples around the origin, confirming the robustness of the method and the reliability of the data (see Fig. 1 B1).

To enhance inter-group separation following the removal of QC samples, we employed supervised Orthogonal partial least squares discriminant analysis (OPLS-DA) to more clearly discriminate samples from the two groups. We observed distinct intra-group aggregation and inter-group separation between the G and the SF diets, as depicted in Fig. 1 B2. The results indicated a noteworthy alteration in the metabolic profiles of yak meat under different feeding patterns. To avoid overfitting of the supervised model during the modeling process, we performed a permutation test to validate its effectiveness. In multivariate statistical supervised modeling, cross-validation with 200 permutations confirmed the reliability of the OPLS-DA model, where R2Y = 0. 797 > 0. 5 and Q2Y = 0. 817 > 0. 5 (Fig. 1 B3). This implies that the initial model has not overfitted, thus confirming its effectiveness for further screening of various metabolites. The OPLS-DA and OPLS-DA loading plot (Fig. 1 B2 and B3) indicated that yak meat produced under different feeding patterns could be distinguished. Supplementing grazing yaks significantly influenced the variation of metabolites in yak meat.

Using VIP values obtained from OPLS-DA (Fig. 1 B4) and P-values from the T-test, we identified significantly different metabolites in yak *longissimus lumborum* muscles between G and SF feeding groups. The significance threshold was set at VIP ≥ 1 and T-test *P* < 0.05. Under this criterion, a positive log2 FC value indicates upregulation, while a negative value indicates downregulation. In the POS mode, we found 177 significantly different metabolites (99 upregulated, 78 downregulated), and in the NEG mode, we identified 198 significantly different metabolites (119 upregulated, 79 downregulated) (Table S3 and Table S4). Additionally, using a Score value > 0.6 as a reliability threshold, we further selected and annotated different metabolites in KEGG metabolic pathways. In both patterns (POS and NEG), a total of 57 annotated significantly different metabolites were identified. After removing duplicates, we obtained 38 annotated significantly different metabolites (Fig. 1 C1, Table 4). Hierarchical cluster analysis (HCA), a widely used unsupervised classification method, was applied to differentiate yak beef samples between the two feeding patterns. As depicted in Fig. 1 C2, the 38 significantly different metabolites accurately classified yak meat based according to the feeding patterns. These 38 metabolites encompass 11 amino acids, 5 organic acids, 5 cofactors, 4 lipids, 3 sugars, 3 purines, 1 peptide, 1 amide, 1 pyrimidine, and 3 other chemicals (Fig. 1 C3). Among them, 31 were upregulated, and 7 were downregulated (Table 4). The log2 FC range for upregulated expression was from 0.44 to 5.37, and for downregulated expression, it was from −0.07 to −1.04.

**Table 4:** Detailed results of the metabolites with the most significant differences in the back muscles of yaks in positive and negative ion detection modes (OPLS-DA VIP > 1 and P value < 0.05)

Metabolite	Molecular weight	m/z	Retention time (s)	VIP	FC	ESI	Direction (Up/Down)	P-value
Pyruvic acid	88.0160	87.01	0.643	2.0853	0.44	NEG	Up	< 0.001
Methylmalonate	116.0726	116.93	1.456	1.3600	0.47	NEG	Up	0.009
L-Tyrosine	181.0739	180.07	0.590	1.0245	0.47	NEG	Up	0.001
L-Isoleucine	131.0946	132.10	0.978	4.0835	0.56	POS	Up	< 0.001
D-Glucose 6-phosphate	260.1051	259.02	0.394	1.6708	0.62	NEG	Up	0.002
L-Tryptophan	204.0899	205.10	1.754	2.2253	0.81	POS	Up	< 0.001
Eicosapentaenoic acid	302.2246	301.22	4.400	1.3346	0.88	NEG	Up	0.003
L-Glutamine	146.0691	147.08	0.309	9.6725	0.91	POS	Up	< 0.001
D-Phenylalanine	165.0790	166.09	1.505	5.2042	0.91	POS	Up	< 0.001
D-Arginine	174.1117	175.12	0.296	2.4735	0.92	POS	Up	< 0.001
Xanthine	152.0334	153.04	0.595	1.1414	0.92	POS	Up	0.043
Arachidonic acid	304.2402	303.23	4.700	4.9290	0.97	NEG	Up	0.002
Epigallocatechin	306.0740	307.08	0.590	1.1936	1.17	POS	Up	0.018
Deoxycholic acid	392.2927	437.29	4.801	1.3988	2.13	NEG	Up	0.035
Pyridoxal phosphate	247.1386	248.15	0.667	10.5088	3.02	POS	Up	< 0.001
Inosine	268.0808	537.17	1.355	2.0633	4.12	POS	Up	< 0.001
Taurine	125.1467	124.01	0.306	11.0795	5.37	NEG	Up	< 0.001
Biochanin A	284.2624	283.25	5.130	1.4188	0.56	NEG	Up	0.002
2-Amino-4-hydroxy-6-(D-erythro −1,2,3-trihydroxypropyl)−7,8-dihydropteridine	331.2707	254.22	4.601	1.1655	0.56	NEG	Up	0.008
Oxaloacetate	132.0564	133.08	0.349	2.2369	0.59	POS	Up	0.003
3-Iodo-L-tyrosine	307.0546	308.09	0.454	7.5651	0.81	POS	Up	0.002
cis-Aconitate	142.0862	173.10	0.327	1.7905	1.10	NEG	Up	< 0.001
N-Formimino-L-aspartate	176.1295	161.14	0.365	2.9003	1.19	POS	Up	0.02
Se-Methyl-L-selenocysteine	196.0578	181.07	0.322	1.4767	1.25	NEG	Up	< 0.001
Cis-8,11,14-Eicosatrienoic acid	306.2559	305.25	4.988	2.5405	1.26	NEG	Up	0.007
L-Fuculose 1-phosphate	210.0828	245.15	0.459	2.7686	1.28	POS	Up	< 0.001
D-Methionine	149.0511	148.04	0.459	1.5093	1.29	NEG	Up	< 0.001
D-Pantothenic acid	219.1107	220.12	1.585	2.0644	1.65	POS	Up	< 0.001
Hypoxanthine	136.0385	137.05	0.453	8.9987	1.66	POS	Up	0.006
D-Arabitol	152.0685	151.06	0.350	1.9067	1.92	NEG	Up	< 0.001
Indoleglycerol phosphate	223.1793	288.22	2.708	2.0825	3.87	POS	Up	< 0.001
Sulfite	80.0632	83.09	6.651	2.4638	−1.04	POS	Down	0.038
Methylselenocysteine Se-oxide	195.9802	199.09	0.293	1.6272	−0.60	POS	Down	< 0.001
L-Citrulline	175.2057	176.10	0.322	1.2699	−0.59	POS	Down	0.001
L-Carnosine	226.1066	227.11	0.296	13.8056	−0.51	POS	Down	< 0.001
L-Glutamyl 5-phosphate	318.1541	226.10	0.344	6.5746	−0.42	NEG	Down	< 0.001
4-Bromocatechol	191.0041	188.00	0.306	1.1516	−0.24	NEG	Down	0.002
Creatine	131.0695	132.08	0.349	10.5846	−0.07	POS	Down	0.040

### Distinguishing yak meat according to two feeding patterns

Linear discriminant analysis (LDA) is a method used to identify groups within diverse samples. In other words, LDA is a technique used to identify group differences within a set of samples (Huang et al., 2021). To obtain the classification and identification results for the conventional G group and SF feeding group, we performed discriminant analysis using the top 5 metabolites with VIP > 1 and notable differences (*P* < 0.05). Two Fisher discriminant functions were used to differentiate yak meats subjected to distinct feeding patterns, outlined as follows:

Y_G_ = 3.697 × L-Glutamine + 11.332 × D-Phenylalanine—182 × Arachidonic acid + 2.329 × Pyridoxal phosphate—19.591 × Taurine—291.049.

Y_SF_ = 4.845 × L-Glutamine + 14.828 × D-Phenylalanine—605 × Arachidonic acid + 3.150 × Pyridoxal phosphate—22.843 × Taurine—530.92.

The established discriminant model was used to categorize beef according to the two feeding patterns, and its efficacy was validated through leave-one-out cross-validation. The outcomes revealed that the model accurately classified 100% of the original grouped cases and 100% of the cross-validated grouped cases (Table 5, Fig. 1 C4). This indicates that supplementary feeding of yaks can significantly affect the concentration of metabolites in yak meat, and the discriminant model established based on these differential metabolites can effectively distinguish yak meat produced according to the two feeding patterns.

**Table 5:** Classification and accuracy of different feeding methods of yaks (confusion matrix) based on the Linear Discriminant Analysis (LDA) model

	Grazing (G)	Supplementary Feeding (SF)	Total
Confusion matrix for original data	6/6	0/0	6/6
	0/0	6/6	6/6
Accuracy for original data	100%/100%	100%/100%	100%/100%
Confusion matrix for cross-validated data	6/6	0/0	6/6
	0/0	6/6	6/6
Accuracy for croos-validated data	100%/100%	100%/100%	100%/100%

### Physicochemical indicators of yak meat according to two feeding patterns

#### Correlation analysis between yak meat physical and sensory quality traits and differential metabolites

**Fig. 2:**
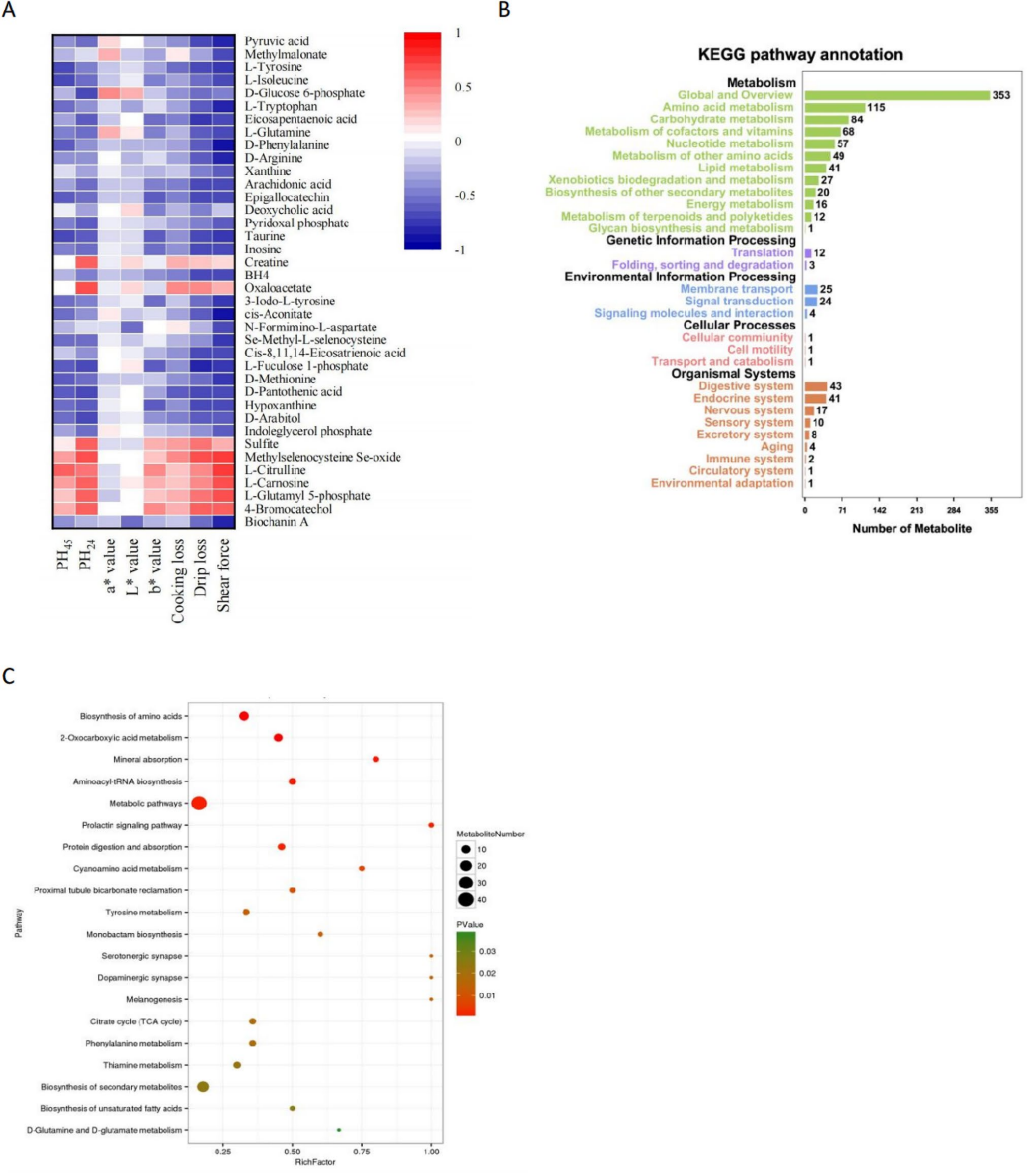
**A** Correlation matrix between yak meat quality parameters and differential metabolites. **B** KEGG statistics of all metabolites in yak meat under two feeding patterns. **C** Top 20 pathways of KO enrichment bubble plot of significantly differential metabolites

As depicted in Fig. 2 a significant correlation exists between 8 edible quality parameters and 38 muscle metabolites. Specifically, parameters such as PH_45_, PH_24_, cooking loss, drip loss, and shear force exhibit positive correlations with levels of some metabolites, including sulfite, methyl selenocysteine Se-oxide, L-citrulline, L-carnosine, L-glutamyl 5-phosphate, 4-bromocatechol, and creatine. Among these, sulfite and methyl selenocysteine Se-oxide are known for their antioxidant properties, which may mitigate oxidative stress in muscle tissues, thereby improving water-holding capacity and meat tenderness. L-citrulline, a key intermediate in nitric oxide metabolism, may regulate blood flow and oxidative balance in muscle tissues, indirectly enhancing meat quality. L-carnosine, with its well-established antioxidant and buffering properties, demonstrate a strong negative correlation with shear force, suggesting its pivotal role in improving tenderness. L-Glutamine 5-phosphate is an important intermediate in protein metabolism, involved in the regulation of amino acid and protein synthesis. In the body, it influences amino acid transport and protein synthesis by binding with glutamate. Specifically, L-glutamine 5-phosphate regulates the balance between protein synthesis and degradation, thus reducing protein breakdown. Reducing protein degradation helps maintain muscle structure and moisture, thereby enhancing the water-holding capacity of meat (Wu et al., 2024). 4-Bromocatechol exerts its effects by regulating antioxidant pathways, reducing oxidative damage in meat, and protecting the fat and protein structures, thereby preserving the flavor and tenderness of the meat. Meanwhile, creatine, as a key molecule in energy metabolism, provides additional energy, delays ATP consumption, maintains pH stability, promotes protein synthesis, and inhibits degradation, improving meat tenderness, water retention, and overall quality. The synergistic effects of both help enhance the structural stability and sensory qualities of meat (Gong et al., 2024; Salau et al., 2020).

In addition, these parameters are negatively correlated with 30 upregulated metabolites, excluding oxaloacetate. This indicates that the upregulation of these metabolites might counteract the improvement of meat quality parameters. Collectively, these 37 significant metabolites, excluding oxaloacetate, have the potential to reduce the five aforementioned meat quality parameters, thus positively influencing overall meat quality. Notably, these metabolites also contribute to the reduction of the b-value (yellowness) of yak meat, which may be linked to their indirect roles in pigment oxidation or antioxidant balance. However, as indicated in Table 3, supplementation does not have a significant effect on the color of yak meat. This lack of significant impact might be attributed to insufficient changes in metabolite concentrations to alter color characteristics or to the fact that meat color is primarily determined by other factors, such as myoglobin content and structure.

#### Differential metabolic pathways in yak meat between grazing and supplemented feeding

To investigate the metabolic pathways influencing yak meat quality under different feeding conditions, we conducted a pathway impact analysis using the Kyoto Encyclopedia of Genes and Genomes (KEGG) for metabolites identified in both positive and negative ion modes. Among the 58 identified metabolites (38 of which showed significant differences), 93 metabolic pathways were implicated (Table S3). Notably, amino acid metabolism and fatty acid metabolism emerged as the most critical contributors to the observed improvements in yak meat quality (Fig. 2B, C).

Amino acid metabolism played a pivotal role in enhancing meat quality, with key pathways such as amino acid biosynthesis, aminoacyl-tRNA biosynthesis, and 2-oxocarboxylic acid metabolism significantly enriched. The amino acid biosynthesis pathway directly influenced protein structure, muscle development, and *post-mortem* proteolysis, which are essential for improving meat tenderness and water-holding capacity. Additionally, the upregulation of aminoacyl-tRNA biosynthesis reflected an increased demand for amino acid incorporation into proteins, facilitating enhanced protein turnover and synthesis under supplemental feeding conditions. This enhanced protein metabolism contributed to improved muscle integrity and reduced cooking loss. Meanwhile, the 2-oxocarboxylic acid metabolism pathway, a critical hub linking amino acid and carbohydrate metabolism, played an essential role in energy production and metabolic flexibility. Enrichment of intermediates such as α-ketoglutarate supported glycolytic flux and maintained *post-mortem* pH stability, directly improving meat tenderness and overall quality. Together, these findings highlight the fundamental importance of amino acid metabolism not only in maintaining muscle structure but also in shaping post-mortem metabolic processes that define meat quality.

Fatty acid metabolism also significantly contributed to the observed improvements in yak meat quality, with pathways related to unsaturated fatty acid biosynthesis prominently enriched. Enhanced synthesis of unsaturated fatty acids improved the nutritional value of the meat while also contributing to its flavor and texture. Key metabolites, including eicosapentaenoic acid (EPA), arachidonic acid, and cis-8,11,14-eicosatrienoic acid, were upregulated and actively participated in pathways such as linoleic acid metabolism, arachidonic acid metabolism, and adipocyte lipolysis regulation. These pathways collectively increased the levels of n-3 polyunsaturated fatty acids (PUFAs) and the PUFA/UFA ratio in yak meat, which are associated with improved health benefits and sensory qualities. Arachidonic acid, an important phospholipid found in meat, liver, and blood, played a crucial role in immune regulation and lipid metabolic cycles, further underscoring the importance of fatty acid metabolism. Furthermore, supplementation significantly increased the concentration of deoxycholic acid, a bile acid involved in lipid digestion, suggesting that supplemental feeding promoted fat digestion and reduced cholesterol synthesis. This observation provides a reasonable explanation for the reduction in cholesterol levels in the supplemented group.

Among the 93 enriched pathways, amino acid metabolism and fatty acid metabolism were identified as the primary drivers of improved yak meat quality under supplemental feeding conditions. Amino acid metabolism enhanced protein synthesis, *post-mortem* metabolic stability, and muscle integrity, while fatty acid metabolism optimized lipid composition, flavor, and nutritional value. Together, these pathways provided key mechanisms for the observed improvements in yak meat quality, demonstrating the potential of targeted feeding strategies to modulate metabolism and improve meat traits.

#### Potential underlying mechanisms

**Fig. 3:**
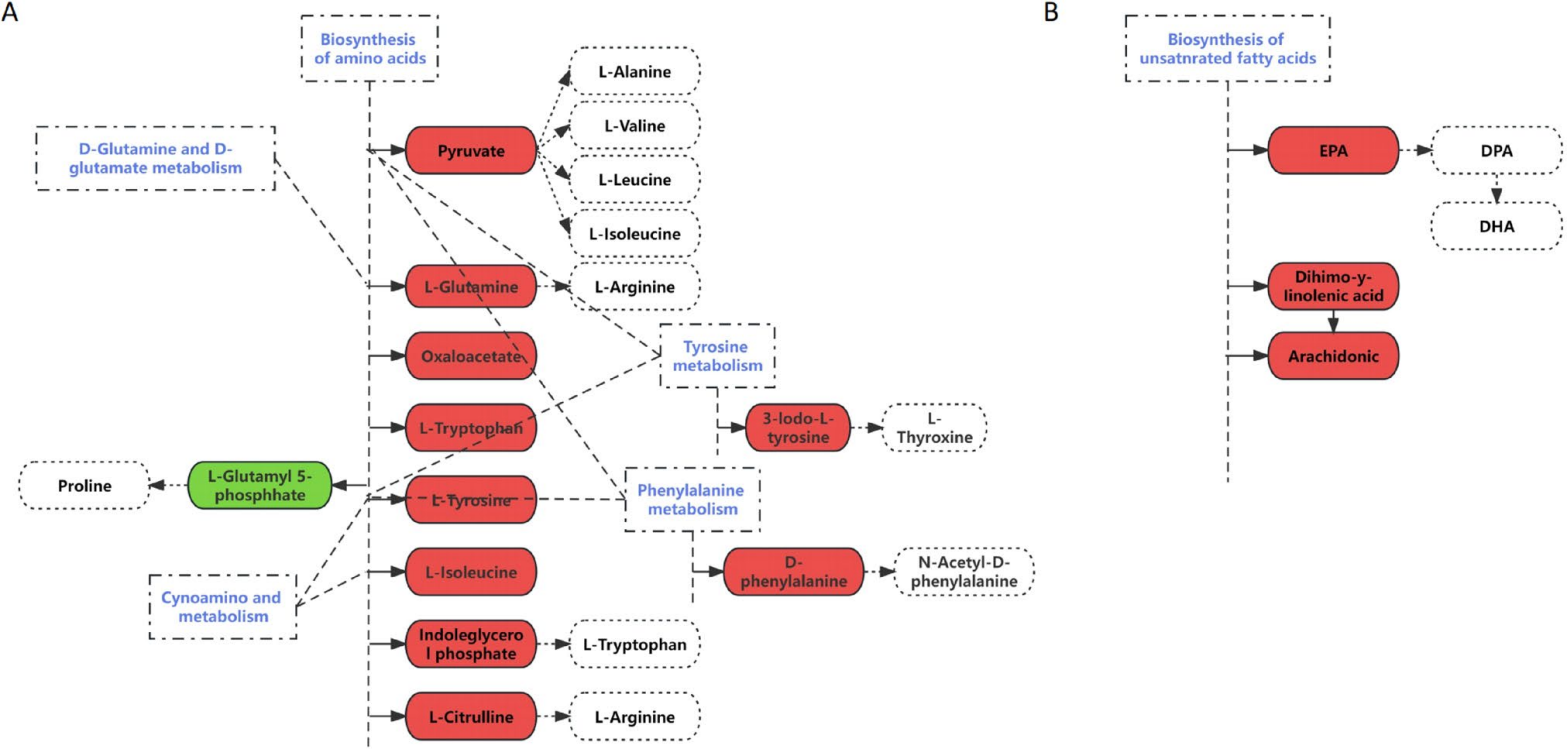
Major metabolic pathways in the *Longissimus lumborum* affected by the diet related to amino acid (**a**) and fatty acid (**b**) metabolism. Pink and green colors represent upregulation and downregulation of metabolites, respectively, while gray indicates the main downstream substances affected by these differential metabolites. EPA, Eicosapentaenoic acid; DPA, Docosapentaenoic acid; DHA, Docosahexaenoic acid. (For interpretation of the color references in the legend of this figure, the reader is invited to consult the web version of this article.)

The observed improvements in yak meat quality under supplemented feeding conditions can be attributed to significant alterations in amino acid metabolism, fatty acid metabolism, and glycolytic pathways, as evidenced by the differential expression of key metabolites. Amino acid metabolism plays a crucial role in this process, influencing protein synthesis, muscle development, and *post-mortem* proteolysis (Reece et al., 2015). In this study, pyruvic acid, L-tyrosine, L-isoleucine, oxaloacetate, L-tryptophan, L-glutamine, and indoleglycerol phosphate were significantly upregulated and actively participated in amino acid biosynthesis (Fig. 3A). These metabolic changes explain the increased essential and total amino acid content observed in the supplemented group, which is closely associated with enhanced meat tenderness and water-holding capacity. For example, L-tyrosine and L-isoleucine are critical for maintaining protein structure and muscle repair, while the enrichment of amino acid biosynthesis and aminoacyl-tRNA biosynthesis pathways reflects an enhanced capacity for protein turnover and synthesis, ultimately improving muscle integrity and reducing cooking loss.

Among these metabolites, guanosine was particularly noteworthy, with its concentration in the supplemented group being 17.4 times higher than in the grazing group. As a key component of RNA, DNA, coenzyme A, and ATP, guanosine promotes cellular energy metabolism and protein synthesis, enhancing liver cell recovery, enzymatic activity, and immune function (Fontecilla-Camps, 2022; Peng & Saito, 2023; Zhang et al., 2022a). These findings indicate that supplementation significantly enhances energy and protein metabolism in grazing yaks, positively impacting overall health and growth performance, consistent with the results of Wang et al., (2018). Additionally, the concentration of toxic sulfites, known to damage cellular structures and DNA and impair growth and development, was markedly reduced in the SF group (*P* < 0.05). This reduction not only improves the nutritional quality of meat but also strengthens the immune response of grazing yaks, further highlighting the benefits of supplementation (Zhang & Mei, 2021).

Fatty acid metabolism also emerged as a critical factor influencing yak meat quality. Key metabolites such as eicosapentaenoic acid (EPA), arachidonic acid, and cis-8,11,14-eicosatrienoic acid were significantly upregulated in the SF group, contributing to metabolic pathways including the biosynthesis of unsaturated fatty acids, linoleic acid metabolism, and arachidonic acid metabolism (Fig. 3B). These metabolic changes were associated with an increased content of n-3 polyunsaturated fatty acids (PUFAs) and a higher PUFA/UFA ratio, both of which are beneficial for meat quality and human health. For example, the elevated levels of EPA and arachidonic acid enhance the flavor and texture of meat by optimizing lipid composition and promoting oxidative stability (Burdge, 2018; Reece et al., 2015). Furthermore, arachidonic acid, an essential phospholipid found in meat, blood, and liver, plays a pivotal role in immune regulation and lipid metabolism, further contributing to improved meat quality (Burdge, 2018). The significant increase in the concentration of deoxycholic acid, a bile acid involved in fat digestion, in the SF group indicates that supplementation promotes fat metabolism while inhibiting cholesterol synthesis (Burdge, 2018; Li et al., 2021). This provides a reasonable explanation for the observed reduction in cholesterol levels in yak meat from the SF group.

In addition to amino acid and fatty acid metabolism, glycolytic activity was also enhanced under supplemented feeding conditions, contributing to the superior tenderness and pH stability of yak meat. Among the identified differential metabolites, D-glucose-6-phosphate was significantly upregulated and actively participated in the pentose phosphate pathway and glycolysis (Fig. 3A). The increased concentration of D-glucose-6-phosphate accelerates the glycolytic pathway, promoting the accumulation of lactate, which stabilizes *post-mortem* pH and prevents excessive acidification, thereby slowing down the hardening of the meat and improving tenderness. At the same time, the elevated unsaturated fatty acid content enhances the distribution and structure of fat in the meat, reducing the rigidity of muscle fibers and further enhancing the tenderness. The combined effects of these two factors provide a comprehensive explanation for the superior tenderness observed in yak meat from the SF group. (Burdge, 2018; Hwang & Joo, 2016).

## Conclusion

This study highlights the significant benefits of supplemental feeding in improving yak meat quality and promoting sustainable grazing systems in high-altitude ecosystems. Supplemental feeding reduced shear force by 39.6%, cooking loss by 8.1%, and drip loss by 9.6%, while increasing PUFA content by 19.6%, EAA content by 16.2%, and the PUFA/SFA ratio by 29.6% (*P* < 0.001). These results demonstrate clear improvements in the physical, chemical, and sensory characteristics of yak meat, making it more appealing to consumers. Mechanistically, these improvements were driven by enhanced amino acid, fatty acid, and glycolytic pathways. Key metabolites, such as L-isoleucine, guanosine, and EPA, contribute to improved protein turnover and lipid stability, thereby enhancing the overall meat quality of the yak. In addition to meat quality, supplemental feeding reduced ecological pressure on natural grasslands, lowering grass consumption by 0.38 kg per yak per day and mitigating overgrazing-induced vegetation degradation.

While this study confirms the effectiveness of UHPLC-QE-MS-based metabolomics in evaluating nutritional impacts, future research should explore the long-term sustainability of feeding strategies and their adaptability across diverse grazing systems. These findings lay the groundwork for developing efficient and sustainable yak production systems, ensuring benefits for both economic and ecological goals.

## Supplementary Information

Below is the link to the electronic supplementary material.

Supplementary file1 (XLSX 1122 KB)

Supplementary file2 (XLSX 818 KB)

Supplementary file3 (XLSX 76 KB)

Supplementary file4 (XLSX 83 KB)

## Data Availability

Data used for this study are available from corresponding author upon reasonable request.

## Abbreviation

UHPLC-QE-MS: Ultra-high-performance liquid chromatography-tandem mass spectrometry
